# Supplementary material for: Performance of a prestressed efficiently prefabricated beam-column connection
Source: PLoS One. 2022 Jul 18;17(7):e0270978. doi: 10.1371/journal.pone.0270978 (PMC9292129; doi:10.1371/journal.pone.0270978)
Supplement: Attachment — Submitted filename: Rebuttal letter in response to reviewers.docx [file pone.0270978.s002.docx]

***Rebuttal letter***

Dear Editors and Referees,

This letter is to notify you that the manuscript has been submitted for consideration of publication in PLOS ONE. The manuscript has neither been published before, nor has it been submitted for consideration of publication in another journal.

At this stage, it is desired to express again sincere appreciation for the comments & suggestions that have been made by the reviewers and Editor considering the initial version of this manuscript. The editor and reviewers’ comments have been taken into careful consideration and the suggested changes have been implemented accordingly. Revised portion are marked in blue in the manuscript.

This paper has been reedited by professional editing, and many language errors have been corrected.

Specifically, the editor and reviewers’ comments are addressed in the following:

**Reviewer #1:**

**Suggestion 1**

- *Section 1.1: please provide more information on the design of the proposed beam-column joints. For example, how the joint is fabricated. How the prestressing is applied. How the energy consuming steel bars are installed. Please also justify the advantages of the proposed joints compared with other forms by others.*

**Answer:** Thank you very much! Your feedback is greatly appreciated. We have described the benefits of the PPEFF joints in the introduction part in lines 57-84. The design information for the PPEFF joints has been re-edited in lines 121-132.

57-84: In the early years, a joint research program of the United States and Japan on prefabricated structures analyzed a hybrid frame system with symmetrical energy-consuming steel bars. The systems had good seismic performance. The research results were incorporated into the design codes of the United States and New Zealand. Due to the complexity of construction and high-quality requirements, the hybrid frame system has not been widely used. When energy-consuming steel bars are installed only in the upper part of the beam, the energy-consuming steel bars near the beam-column interface of the joint are prone to brittle fracture under small displacement. This problem can be solved by applying no bonding treatment of the energy-consuming reinforcement at the beam-column interface. Chinese scholars have investigated beam-column joints with asymmetrical reinforcement. It was found that beam-column joints with prestressed reinforcement installed in the middle of the joint and energy-dissipating steel bars in the upper part of the beam have good energy dissipation capacity and suffer little damage. However, due to the complex structure and the lack of theoretical support for shear design, this configuration has not been widely accepted by the engineering community. The China Construction Group has collaborated with many scholars to optimize the design and developed the PPEFF to promote beam-column joints with asymmetrical reinforcement. Prefabricated laminated beams were used to improve the assembly efficiency of the frame and facilitate the installation of the upper beam reinforcement. The energy-consuming reinforcement was partially unbonded to avoid brittle fracture. In addition, shear reinforcement was installed in the upper part of the beam to meet the requirements of the design code for shear resistance. The research results show that PPEFF joints are more efficient in construction, less environmentally polluting than traditional reinforced concrete beam-column joints, and perform better in terms of initial stiffness, bearing capacity, deformation capacity, and damage conditions. In addition, the prestress of the joints was released to verify their shear resistance; the results indicated an excellent performance.

121-132: The beam was placed flat on the ground to facilitate the fabrication of the large specimen. Ordinary and prestressed reinforcements were used. The prestressed reinforcement was installed in the middle of the specimen, and the energy dissipating bars and the shear reinforcement was located in the upper part of the beam. High-strength mortar was used to attach the beam to the column. The energy dissipating and shear steel bars in the upper part of the beam were then connected using reinforcement links in the column. A 200-mm long polymeric vinyl compounds (PVC) plastic pipe and a section of heat-shrinkable pipe were installed to prevent brittle fracture of the energy-consuming reinforcement near the fixed end of the beam. A hole was located in the middle of the specimen to facilitate the installation of the prestressed reinforcement. A 200-mm thick concrete layer was poured on the upper part of the beam. Fig 3 shows the main fabrication process of the specimens.

**Suggestion 2**

- *It seems that the shear force of the beam is resisted only by the steel bars. Please provide more information on the design of linking bars at the beam-column interface. Whether the steel bars are sufficiently strong and stiff to resist the shear force?*

**Answer:** Thank you! Chinese scholars have tested the shear force resistance of PPEFF joints. In the study, it was found that the energy dissipating and shear reinforcement provided sufficient shear resistance to the PPEFF joints even if the prestressed reinforcement lost their effectiveness, and the contribution of these scholars in this study is mentioned in lines 82-84 of the manuscript.

82-84: In addition, the prestress of the joints was released to verify their shear resistance; the results indicated an excellent performance.

**Suggestion 3**

- *Figure 5: the energy dissipation in the positive loading direction is much larger than in the negative direction, which is due to the use of energy consuming steel bars at the top region of the beam. Please justify such treatment in the text.*

**Answer:** Thank you! Your feedback is a great help to improve the quality of this article. A description of this particular reinforcement arrangement has been added in lines 66-70 of the paper.

66-70: Chinese scholars have investigated beam-column joints with asymmetrical reinforcement. It was found that beam-column joints with prestressing steel bars installed in the middle of the joint and energy-dissipating steel bars in the upper part of the beam have good energy dissipation capacity and suffer little damage.

**Suggestion 4**

- *Section 3.4 is too simple. Please discuss in more detail on the comparison between the predicted results and the test results for the specimens.*

**Answer:** Thank you very much! Your feedback is very important to improve the quality of this article. We have revised this section and placed it in lines 490-501 of the manuscript.

490-501: The comparison of flexural capacity calculated by the equation and obtained from the test is shown in Table 5. On the one hand, the maximum ratio of the calculated results to the test results is 1.26, and the maximum error is 26.5%. Since the strain detection of reinforcement and concrete is easily disturbed by peripheral factors, such as initial cracking of concrete and initial strain caused by the self-weight of the specimen. Therefore, the error between the equation calculation result of SJ-4 and the test result is large, and the calculation error of other specimens is less than 20%. On the other hand, the average error of the specimen samples (0-10) in the yielding stage is 8.07%, and the standard deviation is 7.95%. The mean error of the specimen samples (11-20) in the limit phase is 10.29% with a standard deviation of 5.05%. Generally, the calculated results are in good agreement with the test results. Therefore, the proposed flexural capacity equation is reasonable.

**Reviewer #2:**

**Suggestion 1**

- *This paper is technically sound, but language needs substantial improvement. This reviewer is happy to go through the technical parts after the language is improved. A professional language editing service may be sought.*

**Answer:** Thank you very much! Your feedback was very helpful for the quality of this article. We have extensively revised some of the language expressions in the manuscript and obtained the help of experts in the same field.

**Reviewer #3:**

**Suggestion 1**

- *What do you mean flexural mechanical properties? Did you mean flexural behavior or strength?*

**Answer:** Thank you very much! Your suggestions have helped tremendously to improve the quality of this article. We have reviewed numerous related papers and modified the title of this paper in line 1,2. In addition, the main research work of this manuscript is to study the seismic performance and flexural capacity of PPEFF joints with a view to providing theoretical references for the design of this joints.

1, 2: Performance of a prestressed efficiently prefabricated beam-column connection.

**Suggestion 2**

- *Abstract: The main and important results should be presented.*

**Answer:** Thank you! Your feedback is greatly appreciated. We have revised the summary section of this paper and placed it on lines 14-31.

14-31: Analyzing the seismic performance and flexural capacity of beam-column joints is crucial in structural design phase. The purpose of this paper is to investigate the seismic performance and flexural capacity of precast prestressed efficiently fabricated frame (PPEFF) joints. Reverse cyclic load tests and flexural capacity analysis are conducted. The damage modes, hysteresis curves, skeleton curves, stiffness degradation, ductility, and energy dissipation capacity of five PPEFF joint specimens with different reinforcement rates of the energy-dissipating bars and shear reinforcement are obtained. The results show that the damage pattern of the specimen is ideal, i.e., the plastic hinge region at the end of the beam is severely damaged, whereas the remainder of the beam is slightly damaged. Increasing the reinforcement rate of the energy-consuming steel bars enhances the load capacity, energy dissipation capacity, and initial stiffness of the joint but reduces the ductility performance. The maximum change in ductility was 5.31 for the reinforcement rate of energy-consuming steel bars ranging from 0.38% to 0.59%. In addition, the flexural capacity of the PPEFF joint is evaluated, considering the influence of the shear steel on the yielding and ultimate states. An equation of the flexural capacity is derived. A good agreement is observed between the experimental and calculation results, verifying the correctness of the proposed flexural capacity equation.

**Suggestion 3**

- *Keywords: Abbreviation cannot be used. Please do not use the same words in title and keywords.*

**Answer:** Thank you very much! We have modified the keywords of the manuscript and placed them in lines 33,34.

33, 34: Unbonded prestress, beam-column interface, seismic performance, energy- consuming steel bars, flexural capacity

**Suggestion 4**

- *What is the novelty of this study?! The novelty should be presented in the end of paragraph of introduction. The aim of study should be presented at the first paragraph of methodology.*

**Answer:** Thank you very much! Your feedback is more helpful to improve the quality of this article. We have presented the novelty of this paper in lines 85-91. And the aim of study is given in lines 93-95.

85-91: In this paper, reverse cyclic loading tests were conducted on PPEFF joints with different energy dissipation and shear reinforcement ratios to evaluate the seismic performance and flexural capacity of PPEFF joints. The damage modes, hysteresis curves, skeleton curves, stiffness degradation, ductility, and energy dissipation capacity of the specimens were compared and analyzed. We established a mechanical model of the beam-column interface and derived equations using the plastic hinge theory to calculate the flexural capacity of the PPEFF joints in the yielding and ultimate phases.

93-95: This paper consists of two parts: in the first part, different reinforcement rates for energy-consuming and shear steel bars were used in the reverse cyclic load tests to evaluate the seismic performance of the PPEFF joints.

**Suggestion 5**

- *Did you apply the load as load-control? but why did not you use displacement control? How did you control the applied force of the jack? Did you consider the rate of loading from a test standard like ASTM?*

**Answer:** Thank you very much! Your feedback has been more helpful in improving the quality of this paper. We have revised the description of test loading in lines 158-166 and reviewed Chinese and American standards on seismic proposed static test loading.

158-166: Load and displacement alternate control loading method was used in this test. The axial pressure on the top of the column was constant. The loading procedure is shown in Fig 5. It was divided into two phases. Phase 1: Before the specimen yielded, the design yield load *P_y_* was used as the reference, and one cycle with three load values (0.5*P_y_*, 0.7*P_y_* and *P_y_*) was used. A rate of 5 kg/s was maintained. Phase 2: After the specimen yielded, the yield displacement $\Delta_{y}$ was used. Two loading cycles were completed with an integer multiple of $\Delta_{y}$ and a rate of 0.067 mm/s. The test was stopped when the specimen was loaded to 85% of the maximum load or was destroyed.

**Suggestion 6**

- *The quality of figures, especially in the result, is poor!*

**Answer:** Thank you very much! Your feedback has been more helpful in improving the quality of this paper. We have changed the description of the figures in lines 490-501.

491-502: The comparison of flexural capacity calculated by the equation and obtained from the test is shown in Table 5. On the one hand, the maximum ratio of the calculated results to the test results is 1.26, and the maximum error is 26.5%. Since the strain detection of reinforcement and concrete is easily disturbed by peripheral factors, such as initial cracking of concrete and initial strain caused by the self-weight of the specimen. Therefore, the error between the equation calculation result of SJ-4 and the test result is large, and the calculation error of other specimens is less than 20%. On the other hand, the average error of the specimen samples (0-10) in the yielding stage is 8.07%, and the standard deviation is 7.95%. The mean error of the specimen samples (11-20) in the limit phase is 10.29% with a standard deviation of 5.05%. Generally, the calculated results are in good agreement with the test results. Therefore, the proposed flexural capacity equation is reasonable.

**Suggestion 7**

- *How did you avoid the shear failure in your test setup?*

**Answer:** Thank you very much! Your feedback is more helpful to improve the quality of this paper. In the published paper, tests were done to resist shear damage and it was found that the beams and columns had good shear resistance even with energy dissipation and shear reinforcement alone in case of prestressed reinforcement failure. This part of the description has been added in lines 82-84 of this paper.

82-84: In addition, the prestress of the joints was released to verify their shear resistance; the results indicated an excellent performance.

**Suggestion 8**

- *The literature review can be more comprehensive for example following works on experiments and simulations of reinforced concrete (RC) element could be cited.*

**2020. Performance of fixed beam without interacting bars. Frontiers of Structural and Civil Engineering, 14(5), pp.1180-1195.*

**2019. Performance of a novel bent-up bars system not interacting with concrete. Frontiers of Structural and Civil Engineering, 13(6), pp.1301-1315.*

**Presenting innovative ensemble model for prediction of the load carrying capacity of composite castellated steel beam under fire. In Structures (Vol. 33, pp. 4031-4052). Elsevier.*

**Prediction of the load-carrying capacity of reinforced concrete connections under post-earthquake fire. Journal of Zhejiang University-Science A, 2021, 22(6), pp.441-466.*

**Computational predictions for estimating the maximum deflection of reinforced concrete panels subjected to the blast load. International Journal of Impact Engineering, 2020, 139, p.103527.*

**Surrogate models for the prediction of damage in reinforced concrete tunnels under internal water pressure. Journal of Zhejiang University-SCIENCE A, 22(8), 2021, pp.632-656.*

**Answer:** Thank you! We have read the above article carefully and cite it in this paper.

**Suggestion 9**

- *The theoretical part of the paper can be improved. The background and description of theoretical modes should be better explained. Graphs and flowcharts may help in this section.*

**Answer:** Thank you! We have revised the theoretical part of the paper and added a flowchart of this paper, in line 102.

102: Fig 1. Flowchart of this paper.

**Suggestion 10**

- *Some suggestions for the future works should be proposed in the conclusion.*

**Answer:** Thank you! We have added suggestions for future work in the conclusion section, on lines 536,537.

536, 537: The post-earthquake bearing capacity of the PPEFF joints will be analyzed in-depth in a future study.

**Suggestion 11**

- *The conclusion should be rewritten. The authors should add important results and should explain the summary of their study. Please add the main findings.*

Answer: Thank you! We have re-edited the conclusion, lines 508-537 in the paper.

508-537: Cycling loading tests were conducted on PPEFF joints to analyze the damage modes, hysteresis curves, skeleton curves, stiffness degradation, ductility, and energy dissipation capacity of five specimens. In addition, the effects of different reinforcement rates of the energy dissipating bars and shear reinforcement on the seismic performance of the joints were compared. An equation of the flexural capacity of the joints was proposed. The effect of the shear reinforcement in different force stages was considered in deriving the flexural capacity equation of the PPEFF joints. A mechanical model of the beam-column interface of the joints was established to conduct a force analysis of the reinforcement and concrete. An internal force equilibrium equation and bending moment equilibrium equation were established, and the flexural capacity equations in the yielding and ultimate stages were determined. The findings of this paper are as follows.

1. The test results showed that the damage to all five specimens was beam bending damage. The damage near the plastic hinge region of the beam was more severe, which is consistent with the ideal joint damage mode.

2. Since the energy dissipating bars and shear reinforcement were located in the upper part of the beam, the joint bearing capacity and energy dissipation performance of the beam were higher when the free end moved downward rather than upward, but the ductility was lower.

3. Increasing the reinforcement rate of the energy-consuming and shear steel bars enhanced the energy dissipation capacity of the joint. In addition, decreasing the reinforcement rate of energy dissipating bars enhanced the ductility of the joint. The greatest change in the ductility was observed when the reinforcement rate of energy-consuming bars ranged from 0.38% to 0.59%, with 5.31.

4. A comparison of the calculated and experimental results showed that the average errors in the yielding stage and ultimate stage were 8.07% and 10.29%, respectively, and the standard deviations were small. The good agreement verifies the suitability of the proposed equation, which can be used for the theoretical study of PPEFF joints.

5. The post-earthquake bearing capacity of the PPEFF joints will be analyzed in-depth in a future study.
