# Supplementary material for: Performance of a prestressed efficiently prefabricated beam-column connection
Source: PLoS One. 2022 Jul 18;17(7):e0270978. doi: 10.1371/journal.pone.0270978 (PMC9292129; doi:10.1371/journal.pone.0270978)
Supplement: Attachment — Submitted filename: Rebuttal letter in response to reviewers.docx [file pone.0270978.s003.docx]

***Rebuttal letter***

Dear Editors and Referees,

This letter is to notify you that the manuscript has been submitted for consideration of publication in PLOS ONE. The manuscript has neither been published before, nor has it been submitted for consideration of publication in another journal.

At this stage, it is desired to express again sincere appreciation for the comments & suggestions that have been made by the reviewers and Editor considering the revised version of this manuscript. The editor and reviewers’ comments have been taken into careful consideration and the suggested changes have been implemented accordingly. Revised portion are marked in blue in the manuscript.

This paper has been reedited by professional editing, and many language errors have been corrected.

Specifically, the editor and reviewers’ comments are addressed in the following:

**Reviewer #1:**

**Suggestion 1**

- *It seems that the shear force of the beam is resisted only by the steel bars. Please provide more information on the design of linking bars at the beam-column interface. Whether the steel bars are sufficiently strong and stiff to resist the shear force?*

**Answer:** Thank you very much for your comments. we have added information about beam-column section design in the text, see lines 110-155. We need to explain that the shear force of the PPEFF joint mainly relies on the shear friction between the beam-column interface. In order to meet the requirements of the code "Code for Seismic Design of Buildings" on the shear strength of the beam end, shear reinforcement is arranged in the upper part of the beam. In addition, since ACI 550.3-13 specifies that the shear capacity of energy-consuming reinforcement is not less than the shear capacity of the beam end under constant and live loads [ACI 550.3-13], Chinese scholar (Haishan Guo) investigated the shear performance of PPEFF joints. He relaxed the prestressing force and removed the shear reinforcement before starting the test, and found that the shear capacity of the beam end exceeded the shear capacity of the beam end as specified in ACI 550.3-13 even if it only relied on the suspension chain tensile action of the energy dissipating reinforcement and the direct shear resistance of the prestressing reinforcement, indicating that the PPEFF joints have sufficient shear capacity. In addition, we observed the shear slip at the beam-column interface and found that there was no significant shear slip in the test, which is consistent with the results of Haishan Guo's research.

**Suggestion 2**

- *Figure 5: the energy dissipation in the positive loading direction is much larger than in the negative direction, which is due to the use of energy consuming steel bars at the top region of the beam. Please justify such treatment in the text.*

**Answer:** Thank you very much for your comments. we have added this aspect in the text, see lines 139-153. here we need to explain to you: the asymmetric reinforcement in the beam, i.e. the upper part of the beam is arranged with energy dissipating and shear reinforcement connecting the beam and the column, the longitudinal reinforcement in the lower part of the beam does not extend into the column, the reasons for this design are as follows.

1. When the beam end is subjected to downward load, the beam rotates clockwise, the upper energy dissipating reinforcement is under tension and the lower longitudinal reinforcement is under compression, and this state of force is consistent with ordinary concrete beam-column joints, which have sufficient flexural and shear bearing capacity.
2. When the beam end is loaded upward, the beam rotates counterclockwise, the lower part of the concrete at the beam end is separated from the column, and the upper part of the energy-consuming reinforcement is compressed, resulting in the reduction of the flexural capacity at the beam end. This is conducive to the emergence of the plastic hinge at the beam end in advance, and makes the beam end fully rotate and dissipate energy, and also protects the column end, which is in line with the seismic design concept of "strong column and weak beam" in the seismic design code of the building.
3. Eliminating the connection between the lower longitudinal reinforcement of the beam and the column, this treatment not only saves material cost but also improves the bearing efficiency of the joint. At the same time, the beams can be in the form of composite beams, i.e. the lower part of the beam is precast concrete and the upper part of the beam is cast-in-place concrete. This can improve the efficiency of beam and column installation and reduce the difficulty of construction.

**Reviewer #2:**

**Suggestion 1**

- *The authors are suggested to highlight the scientific importance and novelty of the paper, as this connection may be only suited to a certain type of structures in a certain country, and may lack universality. For example, is PPEFF system used in other countries apart from China?*

**Answer:** Thank you for your comments, which are very helpful to improve the quality of the article, and we have made corresponding changes in the text, see lines 86-97. We need to explain to you that the PPEFF system is evolved from the Hybrid Frame system in the United States and the asymmetric reinforcement strong joints in Japan in the early years, applicable to most of the seismic zones with high seismic intensity. At present, the PPEFF system is mainly used in China for experimental research and engineering practice.

Lines 86-97: At present, researchers have mainly researched the seismic performance of PPEFF joints, but the analysis to quantify the flexural capacity has not been carried out, and there are no corresponding industry guidelines and codes. Therefore, it is necessary to research the flexural capacity of PPEFF joints in order to provide reference for the joints design calculation. In this paper, reverse cyclic loading tests were conducted on PPEFF joints with different energy dissipation and shear reinforcement ratios to evaluate the seismic performance and flexural capacity of PPEFF joints. The damage modes, hysteresis curves, skeleton curves, stiffness degradation, ductility, and energy dissipation capacity of the specimens were compared and analyzed. We established a mechanical model of the beam-column interface and derived equations using the plastic hinge theory to calculate the flexural capacity of the PPEFF joints in the yielding and ultimate phases.

**Suggestion 2**

- *The full name of the term PPEFF should be provided again when it first appears in the main text, even though an explanation has been given in the abstract..*

**Answer:** Thank you! We have modified the text accordingly, see line 36.

Line 36: precast prestressed efficiently fabricated frame (PPEFF)

**Suggestion 3**

- *The basic working principle of a PPEFF connection should be explained before introducing the test program..*

**Answer:** Thank you very much for your feedback. We have made revisions in the text, see lines 139-153.

Lines 139-153: When the specimen is subjected to earthquake, the fixed end of the beam rotates around the column, and the unbonded prestressed reinforcement that maintains the elastic tension state improves the self-resetting capacity, flexural capacity and shear capacity of the specimen. When the free end of the beam is subjected to downward load, the beam rotates clockwise, the energy dissipating reinforcement in the upper part of the beam is under tension and the longitudinal reinforcement in the lower part of the beam is under compression, at which time the beam is in the same state of force as the normal concrete beam-column joint. When the free end of the beam is loaded upward, the beam rotates counterclockwise, the concrete in the lower part of the beam is separated from the column, the longitudinal reinforcement in the bottom part of the beam is not stressed, and the energy dissipating reinforcement in the upper part of the beam is compressed. This beam-column opening mechanism can properly accelerate the emergence of plastic hinge at the beam end, so that the beam end can fully rotate and consume energy to protect the column end, which is in line with the design concept of strong column and weak beam

**Suggestion 4**

- *Any gap opening expected between the beam and the column? The authors may be aware that gap opening mechanism leads to detrimental “beam-growth” or “frame expansion” effect, and as a result severe damage to the slab system. The authors should discuss or at least recognize this issue. For more details about frame-expansion, the authors may refer to (and mention): Experimental and numerical studies on self-centring beam-to-column connections free from frame expansion. Engineering Structures, 198, 109526..*

**Answer:** Thank you! Your comments have been very helpful in improving the content of this paper. We have cited this excellent paper that you recommended in the text. We need to explain to you as follows: when the beam end is subjected to upward load, the beam rotates counterclockwise, and then the concrete below the beam is separated from the column, which is conducive to the emergence of plastic hinge at the end of the beam in advance, so that the beam end can fully rotate and dissipate energy to protect the column end, which is in line with the design concept of "strong column and weak beam" in the seismic design code of the building. In addition, the unbonded prestressing bars in the joints always maintain the elastic tension state, which is conducive to the rapid recovery of the joint after the earthquake and reduces the post-earthquake damage, so the "beam-growth" effect of the joint is small and can even be ignored. In addition, we detected that most of the concrete cracks were closed after the test, and no significant plastic deformation was produced.

**Suggestion 5**

- *The rationale behind the loading protocol (sequence) should be briefly discussed, and the authors should refer to (and mention) the following work: Loading protocols for experimental seismic qualification of members in conventional and emerging steel frames. Earthquake Engineering & Structural Dynamics, 49(2), 155-174, for more details about how a reasonable loading protocol is developed for quasi-static testing of structural members.*

**Answer:** Thank you very much for your suggestion. We have added content in the text in the appropriate places and cited this excellent paper you recommended, see lines 179-186. We need to explain to you that we have referred to the code of seismic building test methods and used quasi-static testing to study the seismic performance of PPEFF joints. In developing the loading protocol, we preloaded the joints in strict accordance with the code and obtained the yield load and yield displacement of the PPEFF joints, and then developed a suitable loading protocol for the PPEFF joints by yield load and yield displacement. The quasi-static testing loading rate was slow, and the adverse effect of loading rate on the stress-strain of the joints was ignored.

Lines 179-186: Reverse cyclic load tests are currently one of the most widely used test methods for structural seismic testing. Static loading is used to simulate the force deformation of a structure subjected to seismic effects as a way to evaluate the seismic performance of the structure. The test has a low loading rate, so its effect on the stress-strain of the structure can be ignored, and it is suitable for seismic analysis under different seismic effects. Therefore, reverse cyclic load tests were used to study the seismic performance of PPEFF nodes, which were loaded according to a mixed control of load and displacement.

**Suggestion 6**

- *The hysteretic loop is not symmetrical. This is a detrimental effect, and may not be accepted by the community of civil engineers. Please comment on this issue.*

**Answer:** Thank you very much! Here we need to explain to you: the asymmetric reinforcement in the beam, i.e., the upper part of the beam is arranged with energy-consuming reinforcement and shear reinforcement extending into the column, while the longitudinal reinforcement in the lower part of the beam does not extend into the column, which is designed for the following reasons.

1. When the beam end is subjected to downward load, the beam rotates clockwise, the upper energy dissipating reinforcement is under tension and the lower longitudinal reinforcement is under compression, and this state of force is consistent with ordinary concrete beam-column joints, which have sufficient flexural and shear bearing capacity.
2. When the beam end is loaded upward, the beam rotates counterclockwise, the lower part of the concrete at the beam end is separated from the column, and the upper part of the energy-consuming reinforcement is compressed, resulting in the reduction of the flexural capacity at the beam end. This is conducive to the emergence of the plastic hinge at the beam end in advance, and makes the beam end fully rotate and dissipate energy, and also protects the column end, which is in line with the seismic design concept of "strong column and weak beam" in the seismic design code of the building.
3. Eliminating the connection between the lower longitudinal reinforcement of the beam and the column, this treatment not only saves material cost but also improves the bearing efficiency of the joint. At the same time, the beams can be in the form of composite beams, i.e. the lower part of the beam is precast concrete and the upper part of the beam is cast-in-place concrete. This can improve the efficiency of beam and column installation and reduce the difficulty of construction.
4. In small earthquake, the constant load can offset the reverse seismic load, so the beam end is generally downward loaded, and the stress state of the beam end at this time is the same as that of ordinary concrete beam-column joints, which means that the joints have "equivalent cast-in-place" energy dissipation capacity. During medium or large earthquakes, the bending moment at the end of the beam generated by the constant load accounts for a smaller proportion of the total beam end bending moment, and the beam end rotates repeatedly with the seismic action. When the beam end rotates counterclockwise around the column, the concrete at the lower part of the beam end is separated from the column, thus reducing the stiffness of the joint, increasing the self-oscillation period, reducing the seismic force, and making the beam fully energy dissipated.

**Reviewer #3:**

- *The manuscript was revised carefully based on the comments. I strongly recommend the paper for publication..*

**Answer:** Thank you very much for your approval of the revised paper, and once again, we express our gratitude to you.
